# Grafting of Poly(ethylene imine) to Silica Nanoparticles for Odor Removal from Recycled Materials

**DOI:** 10.3390/nano12132237

**Published:** 2022-06-29

**Authors:** Sarah Cohen, Itamar Chejanovsky, Ran Yosef Suckeveriene

**Affiliations:** Department of Water Industry Engineering, Kinneret Academic College on the Sea of Galilee, Zemach 1513200, Israel; sarah.cohen00000@gmail.com (S.C.); itamar@kinneret.ac.il (I.C.)

**Keywords:** silica nanoparticles, poly(ethylene imine), ultrasonication, grafting

## Abstract

One of the major obstacles to the reuse of recycled plastic materials is the emanation of after-process odors from recycled polymers and composites. Typically, recycled polymers are blended with an off-odor adsorbent additive in the recycling chain to eliminate these smells. This article describes an innovative ultrasonically assisted method of grafting poly(ethylene imine) (PEI) to silica nanoparticles (SiO_2_) initiated by benzoyl peroxide (BP) which acts as an odor remover. To prepare the PEI/Si, the branched PEI was grafted onto the silica surface without a coupling agent. This made the grafting process straightforward, easy and low in cost. Fourier Transform Infrared (FTIR) analysis confirmed the successful grafting of PEI to silica. The thermogravimetric analysis (TGA) indicated the formation of two different fractions: a polymeric fraction covalently attached to the nanoparticle surface and a non-grafted PEI fraction that was removed during extraction. Up to 30% of the grafted-PEI fractions were produced at the lowest BP concentration with the highest PEI molecular weight at silica-to-PEI weight ratios of (1:1) to (3:1). The sensory assessment showed a substantial reduction in overall odor intensity for 30% of the recycled plastic-containing materials and a ~75% reduction in volatile organic compounds (VOCs) for 100% of the recycled plastics. These results strongly suggest that this innovative PEI/Si nanocomposite can be successfully commercialized for odor removal. To the authors’ best knowledge, this is the first reported work describing a one-pot reaction for grafting PEI to different nanoparticle surfaces.

## 1. Introduction

Plastic waste production worldwide in 2018 was approximately 360 million tons and is expected to rise constantly [1]. Roughly 40% is converted to energy, mainly via combustion reactions. This can be performed by decomposing plastic waste in liquid oil using catalytic pyrolysis as fuel [2]. In this case, only 15% is recycled, and the remainder is dumped in landfills. However, from an economic as well as ecological standpoint, recycling is more appealing, thus making it the leading method of dealing with plastic waste. Although the demand for plastics is growing at a rapid pace [1], raw-material availability is reduced, with increased prices. This underscores the need to produce recycled materials from plastic waste. Packaging waste, in particular from post-consumer use, is the most abundant source of plastic; recycling packaging could contribute enormously to supporting a circular economy [3]. Unfortunately, this type of packaging also has the highest contamination levels which are related to their original applications and can jeopardize their reuse. Huber et al. [4], for example, reported the presence of more than 70 compounds including aromas such as limonene in recycled HDPE originating from household waste.

To endow plastic packaging waste a second life as food or care product packaging or household equipment, the highest standards must be met; thus, the smell and feel of the product must be considered. The most commonly used method to characterize and identify odors is gas chromatography-olfactometry (GC-O). Gas chromatography analysis identifies compounds that cause odors [5], making it possible to link an odor-active component’s molecular structure to its olfactive perception [6]. For example, Strangl et al. [7] investigated the odorant evolution of post-consumer packaging waste during its recycling and reported that during the extrusion stage, the recycled material released a roasted-coffee-bean-like smell that was not characteristic of the original material.

One of main the sources of odor stems from the microbial contamination of the organic matter originally in contact with the plastic waste, as well as the migration of fillers towards the packaging material during storage. This is because plastic materials tend to absorb the scents they are in contact with [8], which imparts an odor to the polymer or alters it as the odorant components degrade [9]. The degradation process of a polymer during its lifetime also contributes to odorant contamination. Specifically, environmental factors such as light, heat and irradiation affect the chemical and physical properties of a polymer through degradation reactions [10,11], causing off-odor emanations [12]. Bravo et al. [13] showed that the thermal oxidation of polyethylene resulted in the release of unpleasant odors which were mainly caused by unsaturated aldehydes and ketones.

In addition, during reprocessing, polymeric materials release odors related to the additives introduced to improve their chemical and physical properties to compensate for previous contaminations and degradations. For instance, a notable odor emission was reported during the reprocessing of LDPE due to the thermal deterioration of antistatic modifying agents [14]. Espert et al. [15] also showed that using natural fiber as a reinforcing agent during the processing of polypropylene induced the formation of low-molecular-weight compounds such as carboxylic acids. Furthermore, polymer degradation itself also contributes to the formation of undesirable odors.

The first step in reducing unpleasant odors is to separate waste at the source. A decrease in the global odor intensity of post-consumer LDPE packaging after sorting was observed via gas chromatography-olfactometry [16]. However, simply cleaning is not effective enough to remove contaminants from recycled polyolefins. More sophisticated approaches need to be considered to improve the quality of recycled polymers. A deep-cleaning process including hot washing and vacuum degassing followed by re-extrusion with melt degassing showed a very high level of contaminant removal for recycled HDPE [17]. Nevertheless, the intensity of the odor was still identifiable in LDPE bags [16]. Masking unwanted odors with fragrances has also been attempted [18], but this method can be ineffective and produce odors such as burning wood and plastic [19].

On the other hand, the use of additives is a powerful technique for odor removal. Sarno et al. [20] designed a graphene aerogel (GA) capable of absorbing a high amount of volatile organic compounds (VOCs) by exploiting the high mesoporosity of GA. Up to 85% and 70%, respectively, of the alcohols and carboxyl acids as well as aldehyde and ketone compounds were absorbed. However, using graphene turns the end-product black.

Zeolite has long been used for VOC removal. Keshavarzi et al. [21] developed a zeolite-based composite film that produced near-undetectable concentrations of ethanethiol and propanethiol. Bugatti et al. [22] reported antimicrobial zeolite-based packaging capable of absorbing phenol and fatty and woody odors.

Alternatively, polycationic polymers such as poly(ethylene imine) (PEI) [23] can be used. Commercial PEIs are mostly branched and possess three different primary, secondary and tertiary amine groups with a theoretical ratio of 1:2:1 [24,25]. Given the growing interest in the high specific surface area of silica (more than 100 of m^2^/g for fumed silica [26]) and its capacity to bind through strong interactions with organic components [27,28], silica-based nanocomposites are widely used [29,30], in particular, PEI-based silica (PEI/Si) [31].

Nanocomposites comprise a new class of materials that is gaining increasing interest. The specificity and functionality of these new nanofillers resides in the fact that even at low loadings, they improve the properties of the polymer, unlike traditional composites [27,29,32].

PEI/Si nanomaterials are powerful absorbents that can remove heavy metals from wastewater due to the high chelation ability of PEI amino groups. Ghoul et al. [33] fabricated PEI-coated silica crosslinked with glutaraldehyde and reported a high percentage of Pb^2+^ and Zn^2+^ ion removal. The crosslinking yielded a larger quantity of attached PEI, but this reduced the absorption efficiency due to the decrease in the percentage of available amino groups. To overcome these limitations, An et al. [34] grafted PEI onto a silica surface via Υ-chloropropyl trimethoxysilane, a coupling agent, and reported that up to 17.5 mg·g^−1^ Pb^2+^ ions were absorbed.

PEI/Si nanomaterials are also good candidates for CO_2_ capture due to their high thermal stability. The most typical method consists of impregnating silica with PEI [35,36,37]. The wide range of properties of PEI/Si nanomaterials make them versatile and potentially applicable to other fields such as biomedicine, including drug delivery and bioimaging, because of the “proton-sponge” effect of PEIs [31]. For instance, mesoporous silica nanoparticles were functionalized through the surface hyperbranching polymerization of PEI, which allowed the latter to be covalently attached to the silica surface [38,39]. Buchman et al. [40] grafted PEI to functionalized-silica nanoparticles using divinyl sulfone as a space link, which has potential applications as a future technique to target cancer cells.

PEI is well known to efficiently remove off-odors [23,41]; however, it is costly and hard to incorporate in polymeric matrices. Associating PEI with silica nanoparticles is a promising approach from both the physical and material points of view. This method makes it considerably easier to incorporate the PEI grafted nanoparticles, since it disperses them better. Furthermore, grafted PEIs have increased surface area, which is crucial for odor adsorption. [42]. Below, we describe an innovative method of grafting PEI to silica nanoparticles that obviates both the need for the usual pretreatment steps and the use of a coupling agent. Briefly, poly(ethylene imine) of different molecular weights (MWs) without further purification was mixed in the presence of a benzoyl peroxide (BP) initiator within a dispersion of ultrasonicated silica particles. The ultrasonic treatment resulted in a uniform dispersion within the polymer matrix, thus preserving the proprieties of the polymer.

## 2. Materials and Methods

### 2.1. Materials

Four commercial poly(ethylene imines) with different MWs were used as received without further purification: PEI 600 and 10K (Alfa Aesar, Tewksbury, MA, USA) and PEI 800 and 25K (D-BASF, Ludwigshafen, Germany). Recrystallized benzoyl peroxide (BP) was used as the initiator (Alfa Aesar, Lancashire, UK). The nanoparticle consisted of fumed silica Aerosil 200 (Si, hydrophilic, 12 nm in size, Evonik, Rheinfelden, Germany). Ethanol (99.9%, Romical, Haifa, Israel) was used as the solvent. Xylene (Carlo Erba Reagents, Val-de-Reuil, France) was used to extract the non-grafted PEI fraction.

### 2.2. Preparation of Nanocomposites

**Figure 1:**
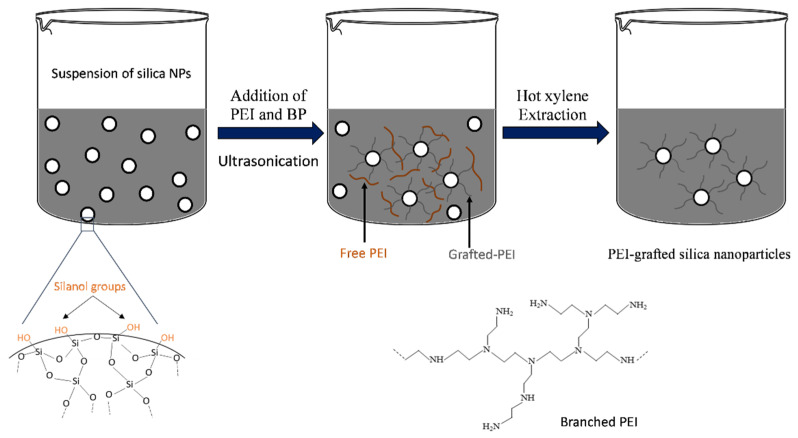
Ultrasonically assisted grafting procedure of the PEI/Si nanocomposites.

PEI/Si nanocomposites are typically synthesized using a one-pot grafting technique, as shown schematically in Figure 1. Here, 0.2 gr of amorphous fumed silica was added to 20 mL of ethanol, followed by sonication for 5 min to disperse the nanoparticles, using an ultrasonic liquid processor (750 Watt Sonicator, Sonics & Materials Inc., Newtown, CT, USA). The sonicator working amplitude was at 25%, and the 0.5-inch probe tip was immersed up to the halfway point of the sample’s volume. The average energy transferred to the reactor was calculated as 200 W/cm^2^. Subsequently, oligomers (PEI 600–800) or polymeric chains (PEI 10–25K) at different weight ratios of Si:PEI (3:1, 1:1 and 1:3) and BP at different weight concentrations with respect to the silica (0.5, 1.0 and 2.0%) were added to the dispersions followed by sonication at 4 °C, as listed in Table 1. The system was left without stirring at room temperature overnight to complete the polymerization. The resulting gel-like nanocomposites were filtrated and washed several times to get rid of the excess ethanol using a Millipore apparatus equipped with a 70 nm diameter membrane. The filtration cake solids were then dried in a vacuum oven overnight, resulting in a fine white powder. The samples were labeled PEIX/Y/Z where X, Y and Z refer to PEI’s MW, weight ratio of Si:PEI and weight concentration of BP:Si, respectively.

Two types of PEI fractions were present in the final solids: free, non-grafted PEI and grafted PEI. To further study the grafting mechanism and its efficiency, an extraction procedure was performed. A defined amount of the solids was immersed in hot xylene for 7 h. The non-grafted PEI was dissolved in xylene, leaving the PEI-grafted nanoparticles at the bottom of the reactor. The resulting nanoparticles were then characterized using FTIR, TGA, HR-SEM and AFM.

### 2.3. Characterization

The thermal stability of the PEI/Si nanocomposites was determined with thermogravimetric analysis (TGA), using a TA Instruments Q5000 Thermal Gravimetric Analyzer (TA Instruments, New Castle, DE, USA). The temperature range was 25–600 °C at a heating rate of 10 °C/min, while monitoring for weight loss as a function of temperature. The analysis was conducted in a nitrogen atmosphere at a flow rate of 25 mL/min.

The Fourier Transform Infrared (FTIR) spectra of the samples were obtained in the range of 650 to 4000 cm^−1^ to determine the chemical composition of the grafted silica nanoparticles using a Thermo 6700 FTIR instrument equipped with a Smart iTR diamond ATR device (Thermo Scientific, Waltham, MA, USA).

The morphology of the nanocomposites before extraction was analyzed using High-Resolution Scanning Electron Microscopy (HR-SEM). The samples were sputtered with carbon prior to observation and examined using an HR-SEM (Carl Zeiss Ultra Plus, Zeiss, Jena, Germany) at an accelerating voltage of 1 keV.

Atomic force microscopic (AFM) measurements were made using an AFM Workshop instrument (SA-AFM, Hilton Head Island, SC, USA) system operated by the non-contact/tapping method of scanning. Our choice of probe was a single-beam cantilever with a force constant of ~40 N/m and a resonance frequency of ~160 kHz. The AFM images were processed using open-source Gwyddion software (Brno, Czech Republic).

## 3. Results and Discussion

### 3.1. FTIR Measurements

**Figure 2:**
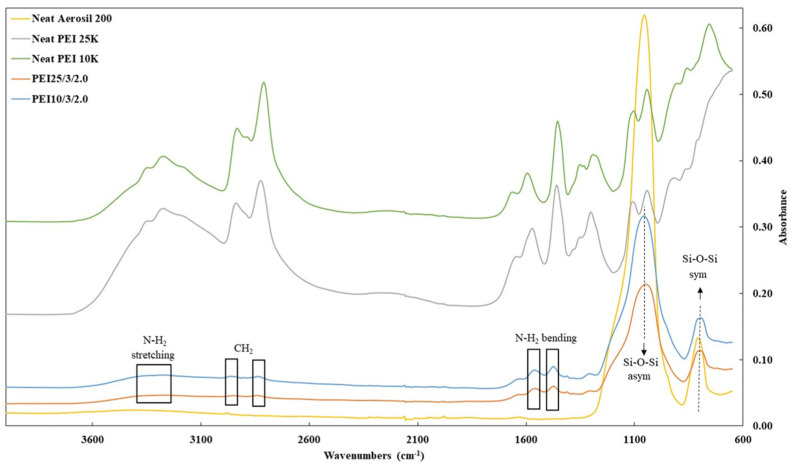
FTIR spectra of pristine silica Aerosil 200 (yellow line), PEI 25K (grey line) and PEI 10 K (green line), PEI25/3/2.0 (orange line) and PEI10/3/2.0 (blue line) before extraction.

FTIR measurements were obtained on the grafted silica particles before and after extraction. All the samples generated highly similar curves independently of the PEI’s MW, Si:PEI weight ratio, and initiator concentration. Figure 2 depicts the FTIR spectra of the pristine Aerosil 200 silica (yellow curve), polymeric chain PEI 25K (grey line) and PEI 10K (green line) and the unextracted samples PEI25/3/2.0 (orange line) and PEI10/3/2.0 (blue line) based on PEI 25K and 10K, respectively. The two peaks characteristic of Aerosil 200 were in the range of 1050–780 cm^−1^ due to the asymmetric–symmetric vibration of the Si-O-Si bond (siloxane groups) [40,43,44] and were clearly present after grafting. The peaks around 1475 cm^−1^ and 1561 cm^−1^ corresponded to the asymmetric and symmetric bending vibration of primary amine N-H_2_ [45], respectively, and the absorption band at ~3280 cm^−1^ reflected the N-H_2_ stretching vibration. In addition, the two peaks around 2820 and 2930 cm^−1^ were clearly characteristic of the C-H stretching vibration from CH_2_ groups [44,45]. These additional peaks thus confirmed the successful grafting of PEI onto the silica surface.

**Figure 3:**
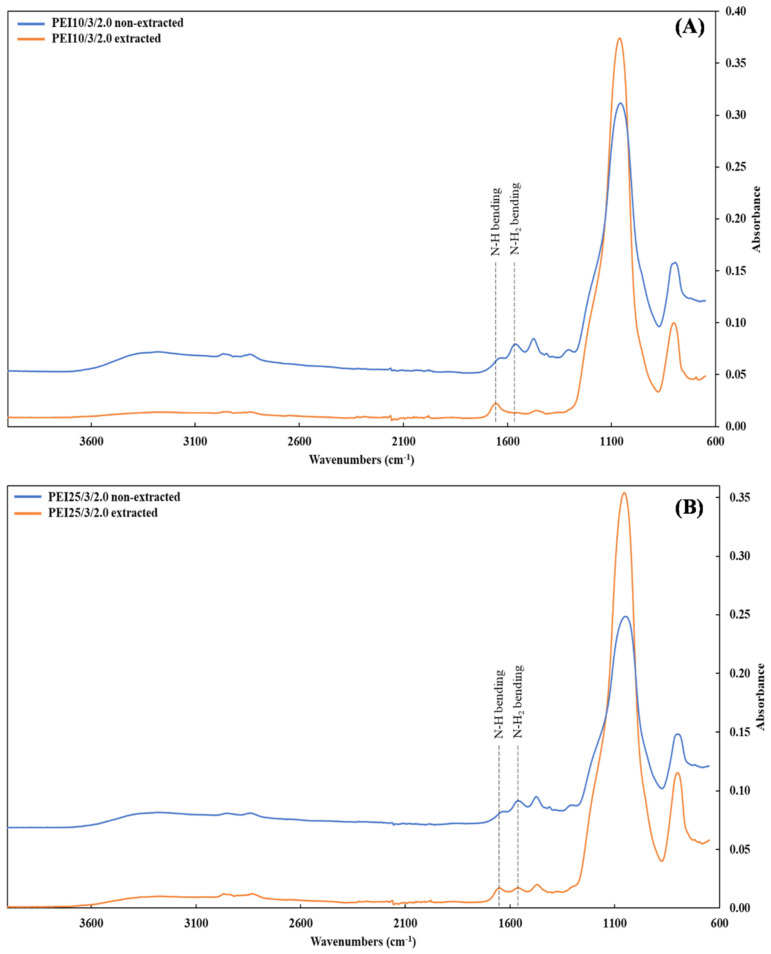
FTIR spectra of the samples (**A**) PEI10/3/2.0 and (**B**) PEI25/3/2.0 before (blue line) and after (orange line) extraction.

**Figure 4:**
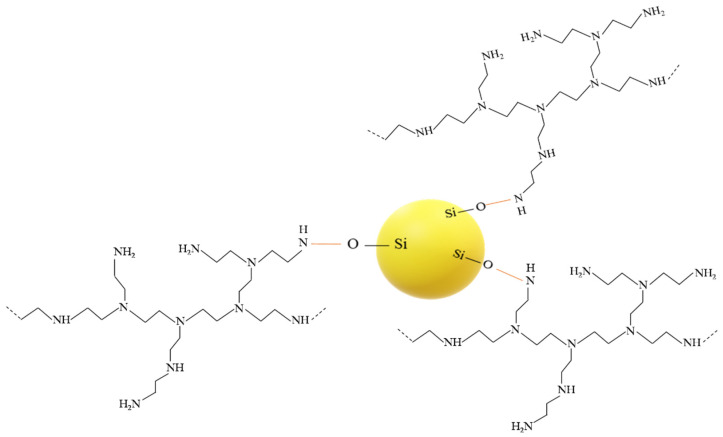
Illustration of chemical bonds between silica and PEI.

After the unattached PEI chains were removed via extraction, the FTIR spectra showed a new peak around 1650 cm^−1^ in both the PEI25/3/2.0 and PEI10/3/2.0, along with the complete or partial disappearance of the peak at 1561 cm^−1^ for PEI10/3/2.0 (Figure 3A) and PEI25/3/2.0 (Figure 3B), respectively. Whereas the peak due to the bending vibration of N-H_2_ (~1561 cm^−1^) underwent considerable intensity reduction, the peak at 1650 cm^−1^ due to the bending of secondary amines N-H [33,45] was heightened by the grafting phenomenon; in other words, most of the primary amines turned into secondary amines. These changes are characteristic of the linkage between the oxygen atoms from the silanol groups of silica and the nitrogen atoms from the PEI [44], resulting in grafted-PEI silica nanoparticles. Figure 4 illustrates the suggested chemical bonds between the PEI primary amine group and the hydroxyl group of silica.

### 3.2. TGA Measurements

**Figure 5:**
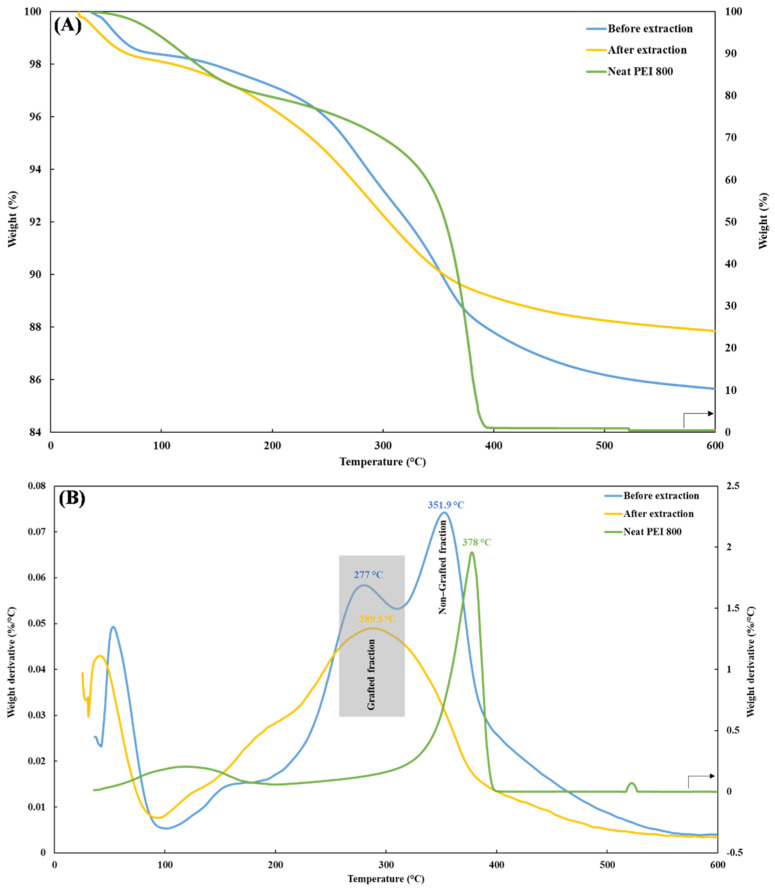
(**A**) TGA and (**B**) DTG thermograms of PEI800/3/2.0 before (blue line) and after (yellow line) extraction and neat PEI 800 (green line).

Figure 5 depicts a typical example of the TGA and DTG (first derivative) thermograms of PEI/Si nanocomposites PEI800/3/2.0, before (blue line) and after (yellow line) extraction. Before extraction, the TGA curve of PEI800/3/2.0 exhibited two weight losses above 200 °C, as confirmed by the presence of two distinct peaks in the DTG curve. The transition below 100 °C was due to the volatilization of the remaining ethanol. After extraction, only one transition remained, and the temperature of the corresponding peak (289.5 °C) was located close to the first peak seen in the unextracted sample (277 °C). In addition, the decomposition temperature of free, neat oligomer PEI 800 (green line) was ~378 °C. Therefore, the first peak in the unextracted sample corresponded to the attached PEI, and the second (at about 351 °C) reflected the free PEI fraction. It might be expected that the latter fraction would degrade first because of its lower molecular weight compared to the grafted-PEI. However, this reverse phenomenon can be better explained by the char barrier effect [30,46], where the nanoparticles form a physical barrier to oxygen transformation and heat insulation, thus protecting the non-grafted PEI. In addition, a temperature difference was observed between the grafted fractions before and after extraction. Similar behavior was previously reported when grafting polystyrene chains to silica and was caused by the effect of chain attachment to the silica surface on the thermal oxidation resistance [29]. Unlike the two-stage decomposition for the unextracted grafted-silica nanoparticles, only one transition was observed after extraction, thus further supporting the claim of successful PEI grafting.

Different grafted-PEI volumes were obtained for different Si:PEI weight ratios and BP concentrations. The reaction parameters (weight ratio, BP concentration and PEI’s molecular weight) were further investigated to determine their influence on the actual grafted-PEI volume.

### 3.3. Influence of the Si:PEI Weight Ratio

**Figure 6:**
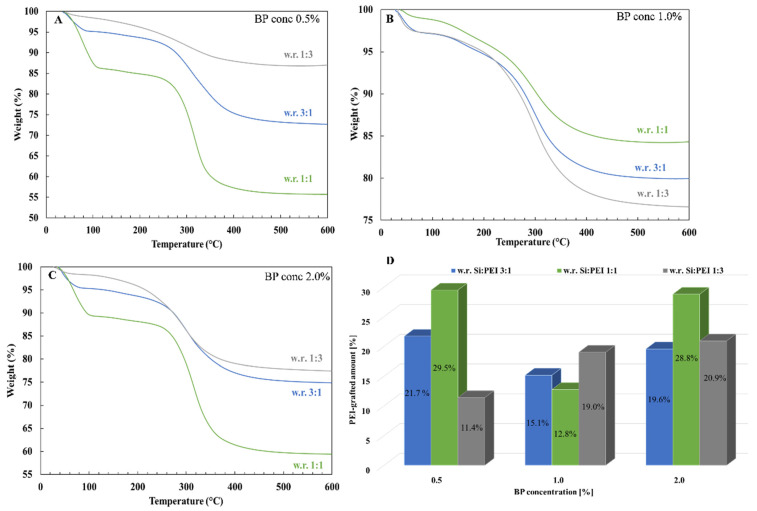
TGA thermograms of the samples PEI25/Y/Z after extraction with a weight ratio 1:3 (gray line), 3:1 (blue line) and 1:1 (green line) for a (**A**) 0.5%, (**B**) 1.0% and (**C**) 2.0% BP concentration and (**D**) histogram of grafted-PEI fractions.

Three different Si:PEI weight ratios (3:1; 1:1 and 1:3) were tested for each BP concentration at three different PEI molecular weights (800–25K). Figure 6 depicts the influence of the weight ratio on the grafted-PEI fraction for the sample series PEI25/Y/Z. For a 0.5% and 2.0% BP concentration, when the amount of PEI was tripled from (3:1) to (1:1), a ~8% enhancement in the PEI-grafted percentage was achieved, whereas an additional increment from (1:1) to (1:3) caused a clear decline in the grafted fraction. This decline suggests that the grafting capacity threshold was reached. Specifically, because of the high branching of PEI 25K, an excessive amount of PEI led to a steric hindrance between the polymer chains, thus physically lowering the PEI’s ability to bind with silica. Nevertheless, for a 1.0% BP concentration, the highest PEI quantity-containing sample (weight ratio 1:3) yielded the greatest attached PEI fraction (19%), whereas for an intermediate amount of PEI (weight ratio 1:1), a lower attached polymeric fraction (~13%) was obtained.

**Figure 7:**
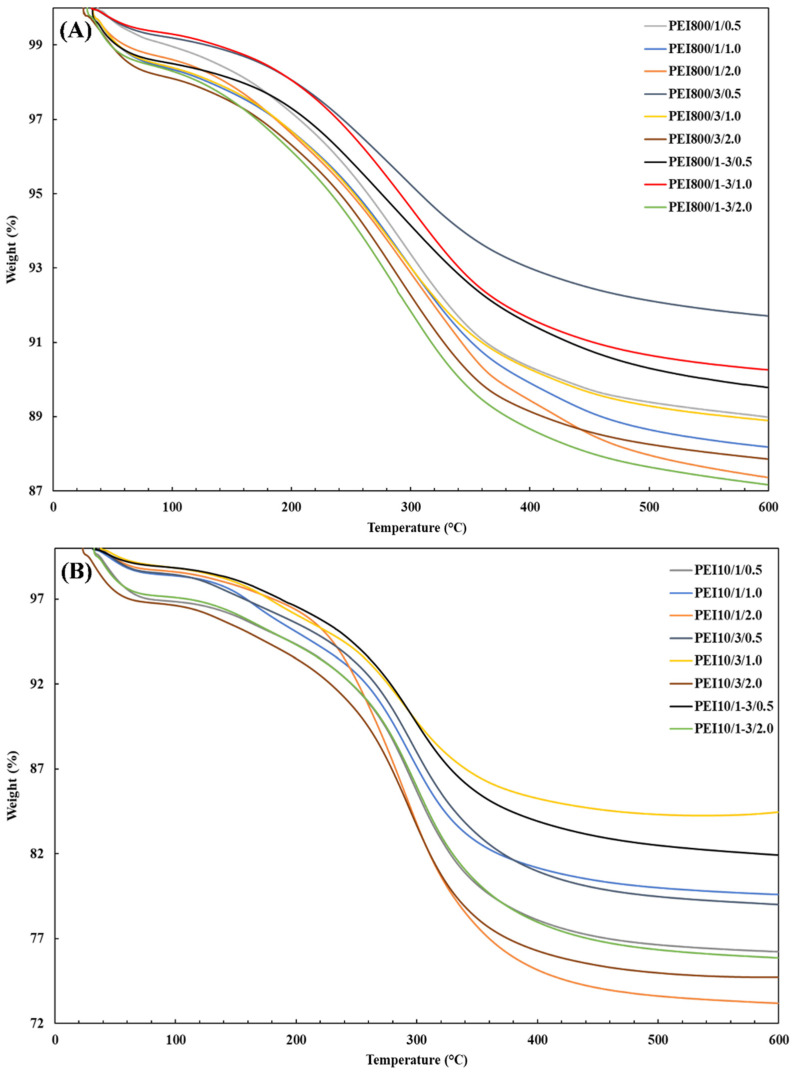
TGA thermograms for the sample series (**A**) PEI800/Y/Z and (**B**) PEI10/Y/Z after extraction.

Figure 7 shows the TGA thermograms for the sample series PEI800/Y/Z and PEI10/Y/Z and confirms the existence of a threshold; the highest polymeric fraction was always obtained for the intermediate weight ratio (PEIX/1/0.5, 1.0 and 2.0). Overall, the PEI800/Y/Z samples did not provide high grafted fractions and were relatively close to each other, with the highest at 11.3%. Greater anchored fractions were achieved with PEI 10K relative to PEI 800 but were still lower than with PEI 25K. Comparatively, 29.5% and 18.6% were grafted to silica for the PEI25/1/0.5 and PEI10/1/0.5 samples, respectively. The attached PEI fraction was hence correlated to the introduced polymer, which was the highest at an intermediate weight ratio.

### 3.4. Influence of the BP Concentration

**Figure 8:**
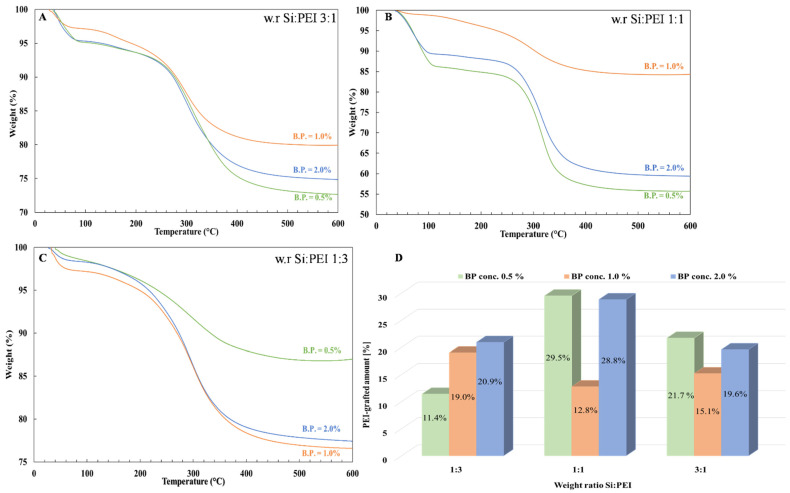
TGA thermograms of the samples PEI25/Y/Z after extraction with a 0.5% (green line), 1.0% (orange line) and 2.0% (blue line) BP concentration for a Si:PEI weight ratio of (**A**) 3:1, (**B**) 1:1 and (**C**) 1:3 and (**D**) histogram of grafted-PEI fractions.

Figure 8 depicts the effect of the initiator’s concentration on the resulting grafted amount for the PEI25/Y/Z samples. For weight ratios (3:1) and (1:1), the grafted fraction was higher at lower concentrations. Interestingly, similar grafting levels were obtained for the highest concentration, indicating that a small amount of BP was sufficient to achieve a large grafted fraction. On the other hand, the lowest grafted fractions were obtained at an intermediate concentration (1.0%). However, for the (1:3) weight ratio, the grafted fraction increased as the concentration increased, although the difference in percentage between the 1.0% and 2.0% BP concentration was small.

In terms of the PEI10/Y/Z samples (Figure 7B), the lowest grafted fractions were also obtained at intermediate concentrations, but in comparison to the PEI25/Y/Z samples, the highest ones were achieved at a higher concentration (2.0%). However, for the PEI800/Y/Z samples (Figure 7A), regardless of weight ratio, the attached oligomeric fraction increased as the BP concentration increased, although only a ~3% augmentation in the grafted-PEI fraction was found. In addition, much lower grafted fractions were obtained with PEI 800, probably because of the lower branching degree of the polymer. The attached PEI fraction to silica was also correlated to the initiator concentration which, overall, was higher at the 0.5% and 2.0% BP concentrations for PEI 25K and PEI 10K, respectively.

### 3.5. Influence of the PEI’s Molecular Weight

**Figure 9:**
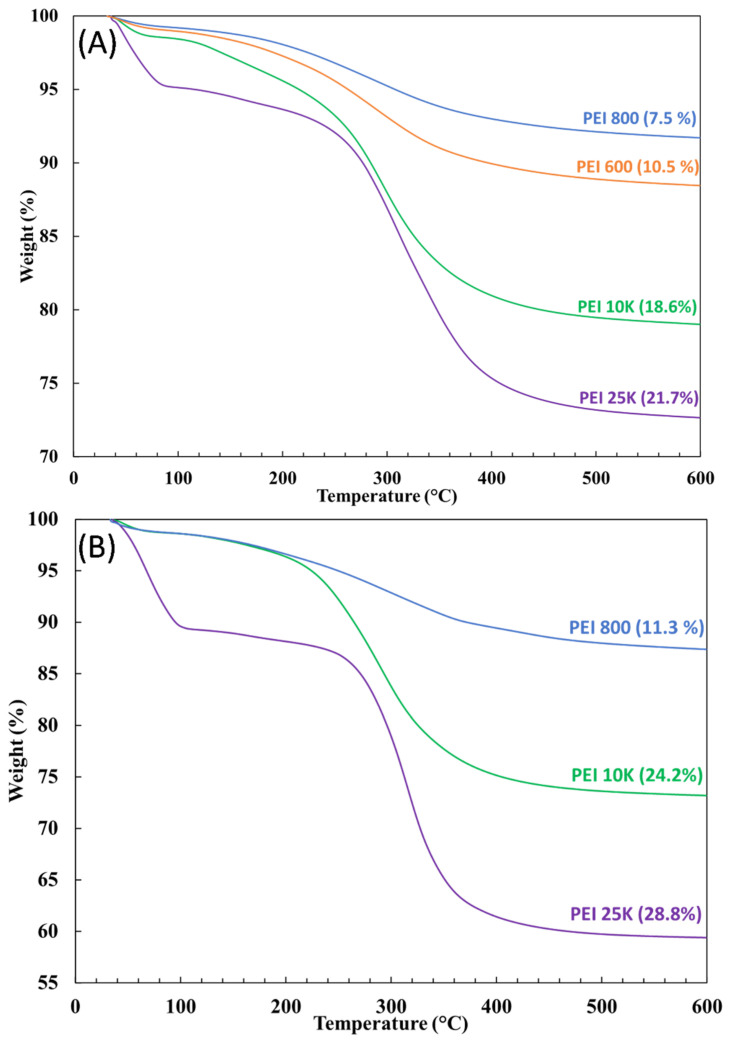
TGA thermograms for the samples (**A**) PEIX/3/0.5 and (**B**) PEIX/1/2.0 after extraction for different molecular weights: PEI 600 (orange line), PEI 800 (blue line), PEI 10K (green line) and PEI 25K (purple line).

As briefly shown above, the polymer’s molecular weight impacted the resulting grafted fraction as well. Examples of TGA thermograms are depicted in Figure 9 for the PEIX/3/0.5 and PEIX/1/2.0 samples at four different molecular weights: PEI 600 (orange line), PEI 800 (blue line), PEI 10K (green line) and PEI 25K (purple line). Regardless of the BP concentration or the weight ratio, the higher grafted fractions were always obtained with higher MW, i.e., PEI 25K and 10K. Because of the greater degree of branching, a larger number of nitrogen atoms were likely to bind with the oxygen atoms from silica [47], resulting in greater effectiveness in the attachment of PEI chains to the silica’s surface.

The PEI/Si nanocomposite selected for further study was PEI25/3/0.5, given that it exhibited the largest grafted fraction (~22%) at the lowest peroxide concentration and weight ratio, thus favoring cost-effectiveness for scale-up.

### 3.6. HR-SEM Measurements

**Figure 10:**
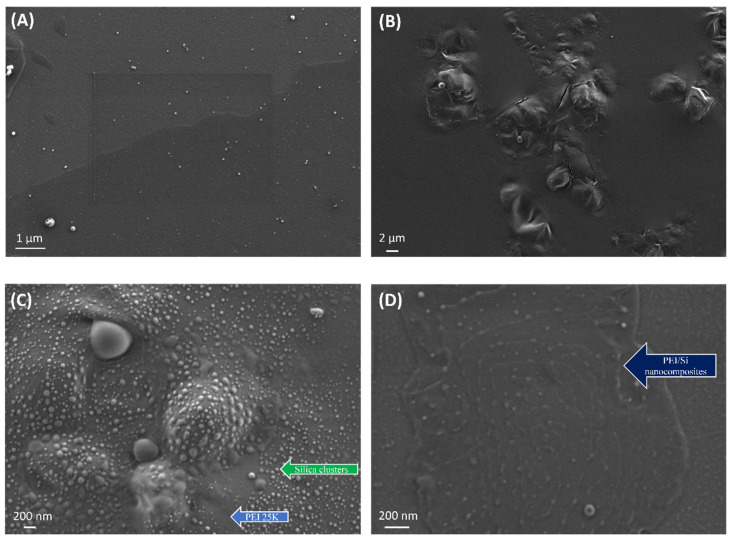
HR-SEM pictures of neat (**A**) silica nanoparticles and (**B**) PEI 25K, (**C**) a physical mixture of silica and PEI 25K and (**D**) PEI25/3/0.5 before extraction.

Figure 10 presents the HR-SEM images of pristine Aerosil 200 silica (Figure 10A) and neat PEI 25K (Figure 10B) as references, as well as a physical Si/PEI mixture (Figure 10C) and the unextracted PEI25/3/0.5 sample (Figure 10D). All the samples were prepared as follows: a defined amount of material was dispersed in toluene, then deposited on a SiO_2_ wafer substrate and placed in the oven for solvent removal. The silica nanoparticles were well-dispersed and produced small agglomerates. By contrast, the neat PEI 25K sample exhibited a considerable homogenous layer with a rough surface. The physical Si/PEI mixture exhibited large silica clusters formed within the polymeric layer. This mixture was initiator-free, without grafting. As seen in Figure 10D, the grafting procedure (unextracted PEI25/3/0.5) resulted in two major differences: the PEI surface became smoother, and the silica nanoparticles were well-dispersed. The longitudinal shapes represent the extended PEI chains wrapping around the Aerosil 200 silica. This confirmed the successful grafting of polymer to silica via the bond formation between the hydroxyl groups of silica and the amino groups of the PEI.

The aggregates (white spots) depicted in the unextracted PEI25/3/0.5 resulted from the presence of free PEI chains that repulsively interacted with each other. This likely engendered the formation of agglomerates. Alternatively, these aggregates may have been formed as a result of the high specific surface area and the hydrophilicity of fumed silica Aerosil 200. The same conclusion was drawn for polyethylene terephthalate/silica nanocomposites [48].

### 3.7. AFM Measurements

**Figure 11:**
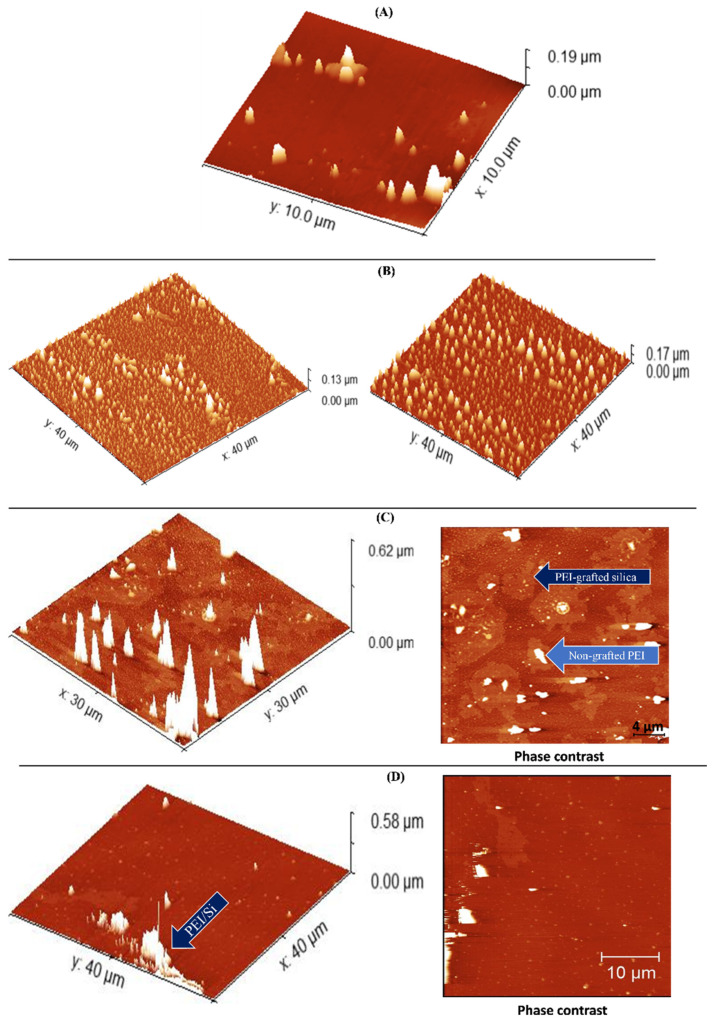
AFM images of neat (**A**) silica nanoparticles and (**B**) PEI 25K and PEI25/3/0.5 (**C**) before and (**D**) after extraction.

Figure 11 depicts the AFM images of pristine Aerosil 200 silica (Figure 11A), neat PEI 25K (Figure 11B) and PEI25/3/0.5 before (Figure 11C) and after (Figure 11D) extraction. All the samples were prepared on a SiO_2_ wafer substrate as previously described. The high homogeneity and roughness of the PEI 25K were confirmed where the height of the free PEI chains was ~150 nm. As seen in Figure 11C,D, the grafting of PEI to silica led to a drop in the roughness along with an increase in the PEI chain height. Before extraction, the height was ~620 nm, whereas, after extraction, it dropped slightly to 580 nm. Figure 11C,D (right side) show the phase contrast before and after free PEI chain removal, respectively, and depict two main regions before extraction—a bright region and a dark region—whereas only one region can be seen after extraction. The bright region can be attributed to compounds with a lower molecular weight corresponding to unattached PEI, whereas the darker region is the result of compounds with a higher molecular weight, thus generating attached PEI chains [49]. The presence of these two phases before extraction confirms the existence of the two different polymeric fractions previously evidenced by TGA: free PEI and grafted-PEI chains. The observation of a single region after extraction suggests that the xylene successfully removed the free PEI, which explains the lesser roughness compared to the unextracted PEI25/3/0.5. In addition, the loss of height observed after extraction suggests a mechanical difference between the free and attached PEI chains. Specifically, because of the attachment to silica nanoparticles, the PEI conformational freedom was restricted in its capacity to move, thus resulting in shorter chains. The topography and phase contrast hence indicated the successful grafting of silica nanoparticles with PEI.

## 4. Conclusions

An original and innovative coupling-agent-free, ultrasound-assisted grafting procedure of PEI polymer onto a silica surface was described for the potential purpose of odor removal. HR-SEM confirmed the successful grafting of PEI to silica through the formation of long chains. The grafting led to two different PEI fractions in the PEI/Si nanocomposites: attached and non-attached PEI chains. The hot xylene extraction resulted in free PEI fraction removal, confirmed under TGA and AFM. In addition, the most branching polymer, i.e., PEI 25K, yielded the higher grafted-PEI fractions, reaching approximately 30%. The functionalized silica nanoparticles were compounded with different polymeric matrices. As described in the supplementary information file (Figures S1 and S2), the incorporation of very low functionalized nanoparticle concentrations (<0.5%) resulted in a dramatic reduction in odor and VOC’s, with a reduction of ~75%.

## Figures and Tables

**Table 1:** Preparation of PEI/Si nanocomposites labeled PEIX/Y/Z; X, Y and Z refer to PEI’s molecular weight (600–25K), the Si:PEI weight ratio and the BP:Si weight concentration, respectively.

BP Conc. [%] → ↓ w.r. Si:PEI	0.5	1.0	2.0
**3:1**	PEIX/3/0.5	PEIX/3/1.0	PEIX/3/2.0
**1:1**	PEIX/1/0.5	PEIX/1/1.0	PEIX/1/2.0
**1:3**	PEIX/1-3/0.5	PEIX/1-3/1.0	PEIX/1-3/2.0

## Data Availability

Suckeveriene, R.Y. Removal of Odors Using Nanocomposites. Provisional Patent Application No. 281,582, 17 March 2021.

## Supplementary Materials

The following supporting information can be downloaded at: https://www.mdpi.com/article/10.3390/nano12132237/s1. Figure S1: Odor panel results for 30% recycled materials; Figure S2: GCMS results for 100% recycled PVC samples reference (gray), 0.5% benchmark (orange) and 0.5% PEI25/3/0.5 (purple).
