# Supplementary material for: Grafting of Poly(ethylene imine) to Silica Nanoparticles for Odor Removal from Recycled Materials
Source: Nanomaterials (Basel). 2022 Jun 29;12(13):2237. doi: 10.3390/nano12132237 (PMC9268616; doi:10.3390/nano12132237)
Supplement: Supplementary file 1 [file nanomaterials-12-02237-s001.zip › nanomaterials-1788102-supplementary.pdf]

nanomaterials

MDPI

Supplementary Materials

Grafting of Poly(Ethylene Imine) to Silica Nanoparticles for Odor Removal from Recycled Materials

Sarah Cohen, Itamar Chejanovsky and Ran Yosef Suckeveriene *

Department of Water Industry Engineering, Kinneret Academic College on the Sea of Galilee, Zemach 1513200, Israel; sarah.cohen00000@gmail.com (S.C.); itamar@kinneret.ac.il (I.C.)
* Correspondence: ransots@gmail.com

Sensory Evaluation of the Recycled Materials

Several polymeric matrices were incorporated within the fabricated functionalized nanoparticles. As a case study for the odor reduction effect, two systems are described herein:

a. Odor Panel

In this study, a 30% w/w post-consumer recycled polymer mixture incorporating low-density polyethylene (LDPE), polypropylene (PP) and grafted maleic anhydride samples were compounded with 70% w/w pristine polypropylene (PP). As a case study, the following samples' odor reduction was studied: i) additive-free recycled material as a reference sample, ii) 2% commercial additive (ZeoFlair 100, ZeoChem, Kentucky, USA), iii) 0.25% neat PEI25 and iv) 0.25% PEI25/3/0.5.

To analyze the odor levels, samples were prepared as follows: 2.5 grams of recycled plastics w/o additives were placed in a 50 ml screw-top glass headspace vial. Then, the sensory evaluation was performed by five pre-trained qualified panelists, based on the German VDA270 standard (Determination of Odour Characteristics of Trim Materials in Motor Vehicles) under 20-25°C. The odor intensity was ranked on a scale from 0 (no noticeable odor) to 5 (unbearable odor), and the average ranking of the panel was recorded.

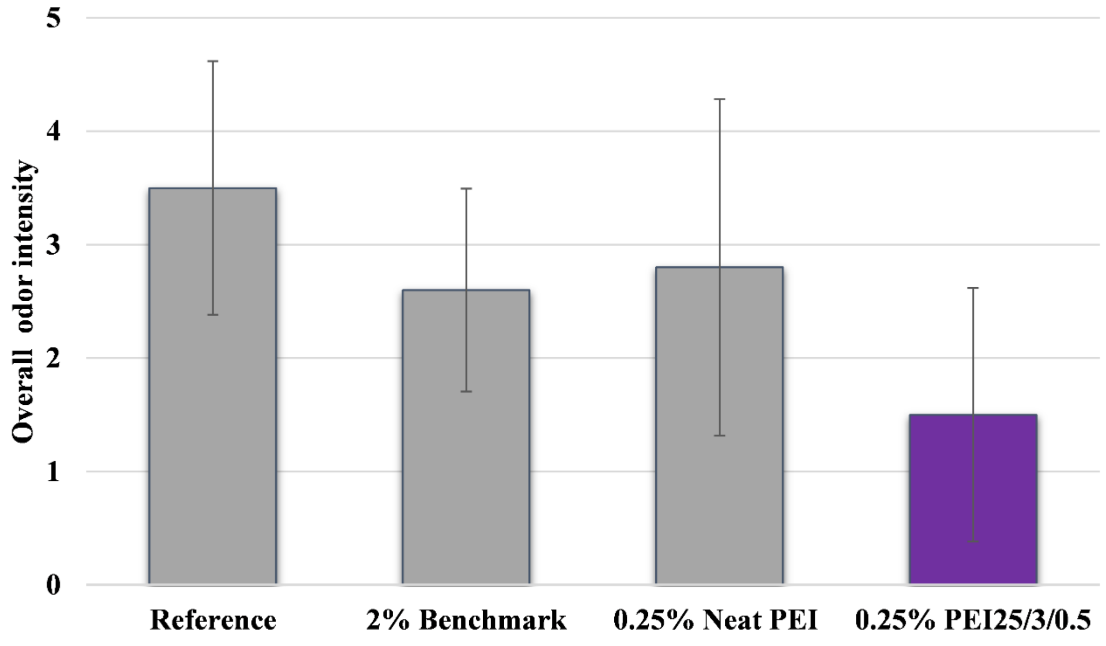

Figure S1. Odor panel results for 30% recycled materials.

b. GCMS Analysis

In this study, 100% recycled poly(vinyl chloride) was compounded with 0.5% w/w odor removal commercial additive (Struktol RP-53, Struktol company of America LLC, Ohio, USA) and 0.5% w/w PEI25/3/0.5 functionalized nanoparticles. The samples were analyzed using a Clarus 680 (Perkin Elmer Inc., USA) gas chromatograph (GC) equipped with a Clarus SQ8T (Perkin Elmer Inc., USA) mass spectrometer (MS). Each sample was analyzed at 90°C for 30 minutes and at 120°C for 60 minutes. The total amount of volatiles was recorded. Figure S2 depicts the total number of volatiles obtained from each sample. It is clear that the reference and the sample containing the commercial additive are practically the same, showing no evidence of odor removal. However, the samples containing PEI25/3/0.5 showed a dramatic reduction of 70-75%, compared with the reference PVC. Thus, one can conclude that even a low load of the functionalized nanoparticles has a great impact.

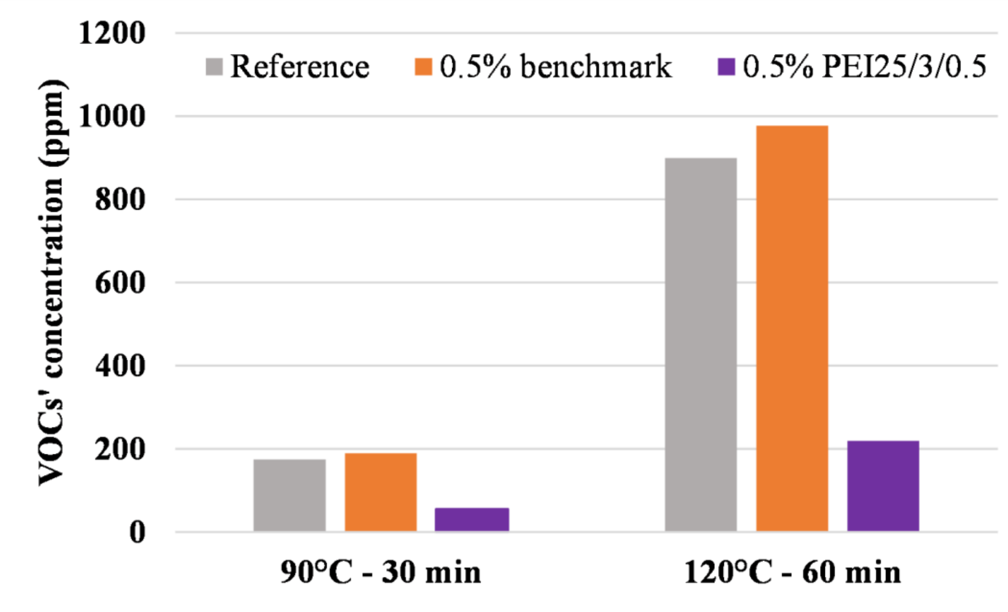

Figure S2. GCMS results for 100% recycled PVC samples reference (gray), 0.5% benchmark (orange) and 0.5% PEI25/3/0.5 (purple).
